# The race for antimicrobials in the multidrug resistance era

**DOI:** 10.1111/1751-7915.12884

**Published:** 2017-12-04

**Authors:** Carlos Molina‐Santiago, Antonio de Vicente, Diego Romero

**Affiliations:** ^1^ Departamento de Microbiología Instituto de Hortofruticultura Subtropical y Mediterránea “La Mayora” Universidad de Málaga Bulevar Louis Pasteur 31 (Campus Universitario de teatinos) 29071 Málaga Spain

## Abstract

The appearance of multidrug‐resistant pathogens is a major threat to human health with the reemergence of fatal and untreatable diseases. In addition to a rational use of the well‐known and available antibiotics, two complementary ways to overcome this public health issue are (i) the discovery of new antimicrobials and (ii) the chemical modification of pre‐existing potent antibiotics. In this article, we highlight some of the strategies to generate new and promising antimicrobials for use in the management of these so‐called ‘superbugs’.

## Highlight

The discovery of penicillin by A. Fleming in 1928 (Fleming, 1929) marked a milestone in the history of medicine. Penicillin served to treat infections caused by *Streptococcus* and *Staphylococcus* and had great relevance in the victory of the Allied forces during the Second World War. The isolation of streptomycin to combat tuberculosis infections, the large‐scale production of antibiotics and the discoveries of the main families of antimicrobials used today are additional achievements that defined the so‐called Golden Age of Antibiotics (Comroe, 1978). Since then, the discovery of novel natural antimicrobials has dramatically decreased, and structural modifications to the backbone of some of the well‐known and more potent natural compounds have become the driving force for the diversification of the battery of molecules to be used in the treatment of infectious diseases. However, different social and economic issues, along with the evolution and widespread distribution of antibiotic‐resistance elements in bacterial pathogens, have compromised the eradication of neglected or easily treatable diseases (Brown and Wright, 2016). Indeed, a devastating report by the World Health Organization (WHO) warned of the exponential increase in the death rate in patients infected with multidrug‐resistant pathogenic bacteria, fundamentally due to the ability of these strains to become resistant to the last lines of antibiotics (Taconelli and Magrini, 2017) (http://www.who.int/medicines/publications/WHO-PPL-Short_Summary_25Feb-ET_NM_WHO.pdf?ua=1). Examples include *Pseudomonas aeruginosa* or *Acinetobacter baumannii* strains that are responsible for multiple nosocomial diseases and are resistant to β‐lactams, such as carbapenems or third‐generation cephalosporins (Lister *et al*., 2009; Vila and Pachon, 2011). Intensification of financial incentives, new regulatory criteria for drug approvals and the identification of new therapeutic targets for antibiotic discovery are actions to be taken to overcome this threat to public health (Laxminarayan, 2014).

In the last few decades, creative experimental approaches have been developed, which either alone or combined, are starting to offer satisfying and promising results. One illustrative example is the development and use of iChip technology (Nichols *et al*., 2010) that permits the growth of uncultured microorganisms, which along with directed screenings against bacterial pathogens, have led to the identification of teixobactin, a novel antibiotic produced by the recently isolated *Eleftheria terrae* bacterium. This molecule binds to a highly conserved motif of lipid II and lipid III, arresting the cell wall synthesis (Ling *et al*., 2015). The authors describe and propose teixobactin as the first member of a new class of antibiotics that has evolved to minimize the risk of resistance development, a fact supported by the absence of resistant strains of *Mycobacterium tuberculosis* or *Staphylococcus aureus* in long‐term experiments (Ling *et al*., 2015). The discovery made by Ling and collaborators also proves that the environment is still a poorly exploited source of new antimicrobials and highlights the need for novel technologies to decrease our dependence on cultivable methods in the race for the discovery of new active molecules against multidrug‐resistant pathogens (Ling *et al*., 2015). Many other groups have also applied this fundamental idea to the screening of diverse environments for potential novel antibiotics with specific targets. Maffioli and collaborators screened different soil‐microbial libraries and identified the compound pseudouridimycin (PUM), the first nucleoside analog that selectively inhibits the bacterial RNA polymerases (RNAP) (Maffioli *et al*., 2017). Interestingly, PUM did not show cross‐resistance with current antibiotics and exhibited low rates of resistance emergence, two critical features that make PUM a very promising antibiotic for clinical purposes. These two new antibiotics are examples of the outstanding potential of soils as resources waiting to be mined for the discovery of new antimicrobials.

As stated above, chemical modifications of already‐discovered antibiotics became prevalent in the antibiotic discovery field, a strategy that replenished the arsenal of antibiotics by semisynthetic and, to a lesser degree, fully synthetic routes. Despite the loss of efficiency of most of these modified molecules due to the development of resistance, a recent report by Okano and collaborators highlighted the great potential that chemical modification still has to generate novel molecules with low risks of the emergence of resistance (Okano *et al*., 2017). To illustrate this idea, the authors focus on vancomycin, a last‐line antibiotic in clinics. The diversity of the modes of action of vancomycin, and therefore the need of pathogens to accumulate mutations in different targets to become resistant, is likely behind the delay in the emergence of resistant strains over more than 60 years of use (Rubinstein and Keynan, 2014). However, vancomycin‐resistant pathogenic strains have evolved, probably due to the incorporation of the vancomycin‐resistant mechanisms from vancomycin‐producing bacteria (Marshall *et al*., 1998).

The conversion of *D*‐Ala‐*D*‐Ala to *D*‐Ala‐*D*‐Lac in the peptidoglycan termini is one of the mutations that provide resistance to native vancomycin, however, chemical modifications directed to key single‐atom sites in the binding pocket of vancomycin seem to enable the antibiotic to bind both the modified and the unaltered targets in the peptidoglycan (Crowley and Boger, 2006; Xie *et al*., 2012; Okano *et al*., 2015). Peripheral structural modifications, such as a (4‐chlorobiphenyl) methyl (CBP) group, increased the antimicrobial potency of vancomycin and provided, with multiple synergistic mechanisms of action, a delay in the emergence of resistance (Okano *et al*., 2014, 2015). This chemical modification served as a starting point for the introduction of further single peripheral modifications at the C‐terminus, which led to a battery of new and improved versions of vancomycin against a vancomycin‐resistant pathogen (VanA VRE). The incorporation of a quaternary ammonium salt bearing a tetradecyl substituent, called C14, resulted in an increase in the antimicrobial potency 10‐ or 1000‐fold higher than the vancomycin derivative or native vancomycin, respectively, a gain apparently related to membrane permeability as a second mode of action (Okano *et al*., 2017). Another peripheral modification of the CBP‐vancomycin derivative, called C1 (quaternary ammonium salt bearing a methyl substituent), generated a new version, displaying three modes of action that cooperatively arrest the cell wall synthesis: (i) a pocket modification with affinity for both *D*‐Ala‐*D*‐Ala and *D*‐Ala‐*D*‐Lac bonds and the ability to inhibit the transpeptidase‐catalyzed cross‐linking; (ii) a CBP disaccharide modification that enables the direct inhibition of the transglycosylase reaction without the need to bind to *D*‐Ala‐*D*‐Ala/*D*‐Ala‐*D*‐Lac; and (iii) the C1 quaternary ammonium salt C‐terminal modification responsible for membrane permeability. The result is a modified vancomycin molecule with the most potent inhibition of cell wall synthesis and the most pronounced and potent induction of cell membrane permeability, of all the compounds examined to date, with up to 10 000‐fold more potency than the original vancomycin (Okano *et al*., 2017). Furthermore, the fact that there were no changes in the susceptibility of diverse pathogenic bacteria to this new version of vancomycin after up to 50 days of experiments, indicated that the combination of the three different mechanisms of action complicates an otherwise easy evolution of pathogenic bacteria from sensitive to resistant.

In summary, the discovery of new antibiotics to address multidrug pathogenic bacteria is critical for human health, and our ability to be successful requires novel and creative screening strategies along with economic support and social awareness. New experimental and screening approaches are promisingly leading to the discovery of new antimicrobial compounds such as teixobactin and pseudouridimycin. On the other hand, as illustrated with vancomycin, well‐established chemical modification protocols may alternatively contribute to the diversification and strengthening of the potency of natural molecules. Finally, the two strategies together are foreseen to expand our capacity to generate novel and more successful molecules for the treatment of diseases caused by multidrug‐resistant bacteria.

## Conflict of interest

None declared.
